# Cyanotoxins in Bloom: Ever-Increasing Occurrence and Global Distribution of Freshwater Cyanotoxins from Planktic and Benthic Cyanobacteria

**DOI:** 10.3390/toxins14040264

**Published:** 2022-04-08

**Authors:** Triantafyllos Kaloudis, Anastasia Hiskia, Theodoros M. Triantis

**Affiliations:** 1 Laboratory of Organic Micropollutants, Water Quality Control Department, Athens Water Supply & Sewerage Company, EYDAP SA, Flias 11, 13674 Menidi, Greece; 2 Institute of Nanoscience and Nanotechnology, National Center for Scientific Research “DEMOKRITOS”, Partiarchi Grigoriou E & Neapoleos 27 Str., Agia Paraskevi, 15341 Athens, Greece; a.hiskia@inn.demokritos.gr (A.H.); t.triantis@inn.demokritos.gr (T.M.T.)

Toxic cyanobacteria in freshwater bodies constitute a major threat to public health and aquatic ecosystems [1]. Cyanobacterial blooms are increasing in frequency, magnitude and duration globally, while eutrophication, rising CO_2_ and climate change promote their global expansion [2,3]. Toxic cyanobacteria metabolites, known as cyanotoxins, comprise a wide range of compounds, including cyclic peptides (microcystins, nodularins) and alkaloids (cylindrospermopsins, anatoxins, saxitoxins) that can be hepatotoxic, cytotoxic, genotoxic or neurotoxic. In response to the risks associated to known cyanotoxins, the World Health Organization (WHO) has published guidelines for their monitoring and management, including provisional guideline values for exposure via drinking water and recreational activities [4]. Nevertheless, the high metabolic potential of cyanobacteria yields a plethora of secondary metabolites that are largely understudied. A recently developed database of cyano-metabolites reported in the literature (CyanoMetDB) contains more than 2000 molecules, including more than 300 microcystin congeners [5]. Still, research so far on the occurrence and impacts of cyano-metabolites has mostly focused on a small number of cyanotoxins, particularly on a few microcystin congeners.

This Special Issue aims to present novel research results on the presence and structural diversity of cyanotoxins and cyano-metabolites in freshwater bodies worldwide. We welcomed research and review papers that showcase the expanding global geographical spread of cyanotoxins, including reports from less-studied areas and on understudied cyanotoxins and cyano-metabolites. We particularly encouraged advances and novelties in the areas of cyanotoxin analysis and monitoring, structural elucidation of new cyano-metabolites, biotic and abiotic factors linked to cyanotoxin production and the role of benthic cyanobacteria as cyanotoxin producers. 

A number of published papers reported the presence of toxic cyanobacteria and cyanotoxins using diverse monitoring techniques, in freshwater bodies encompassing Central and South European, Mediterranean, Southeast Asian and North American regions. Van Hassel et al. [6] reported results from monitoring of cyanobacterial blooms in lakes of Wallonia, Flanders and Brussels, Belgium, using LC-MS/MS, PCR and sequencing techniques, to assess the risks associated to recreational waters. More than 20% of samples exceeded the WHO guideline value for microcystins, while the *mcyE* gene was detected in 76% of samples. Fournier et al. [7] investigated the deep-water, red-pigmented biomass occurrences in Lake Constance, which is the third largest lake in Central-Western Europe that borders Germany, Austria and Switzerland. Using 16S rRNA gene-amplicon sequencing and LC-MS/MS they showed that these blooms were contributed by microcystin-producing *Planktothrix* spp. A one-year monitoring study of Slovenian waterbodies using qPCR (*mcyE*, *cyrJ*, *sxtA* genes) and LC-MS/MS (microcystins, cylindrospermopsin, saxitoxin) was conducted by Zupancic et al. [8]. Potentially toxic *Microcystis* and *Planktothrix* cells were detected by qPCR and microscopic analysis and a positive correlation between the numbers of *mcyE* gene copies and microcystin concentrations was observed. Furthermore, potential cylindrospermopsin and saxitoxin producers were detected by qPCR, showing the potential of molecular techniques to complement chemical and microscopic analysis in freshwater monitoring programs. Zervou et al. [9] reported the results of a 3-year monitoring of Lake Vegoritis in Northwestern Greece, which is used for irrigation, fishing and recreational activities. LC-MS/MS analysis showed the co-occurrence of cyanotoxins (seven microcystin congeners and cylindrspermopsin, at low levels, <1 μg/L) with other cyanobacterial peptides (anabaenopeptins, microginins). An investigation for the presence of cyanotoxins in Lake Karaoun, the largest artificial lake in Lebanon that serves multiple purposes, was conducted by Hammoud et al. [10], using complementary analytical techniques (LC-MS/MS, qPCR, ELISA and in vitro bioassays). A total of 11 microcystin congeners were detected in concentrations up to 211 and 199 μg/L for MC-LR and MC-YR, respectively. In addition, typical volatile and odorous cyanobacteria compounds were detected by GC-MS. Using a polyphasic approach, Ballot et al. [11] characterized cyanobacterial strains isolated from Meiktila Lake, a shallow reservoir close to Meiktila city in central Myanmar. The strains were classified morphologically and phylogenetically as *R. raciborskii*, and *Microcystis* spp. Cylindrospermopsins were detected by ELISA and LC–MS in 3 of the 5 *Raphidiopsis* strains, while *Microcystis* strains produced a wide range of microcystins, including 22 previously unreported congeners. Zastepa et al. [12] characterized nearshore deep chlorophyl layers from two embayments of Lake Huron, Canada. These layers were shown to be dominated by *Planktothrix* cf. *isothrix.* Microcystins, anabaenopeptins and cyanopeptolins were detected through the water column, along with the corresponding genes. The results also indicated that intersecting gradients of light and nutrient-enriched hypoxic hypolimnia are key factors in supporting deep chlorophyl layers in these embayments.

A serious incident involving dog deaths in Mandichosee, a mesotrophic reservoir of the River Lech, Germany, was investigated by Bauer et al. [13]. Anatoxin-a and dihydroanatoxin-a (dhATX) from benthic *Tychonema* sp. were detected by LC-MS/MS in the stomachs of two dogs in concentrations up to 1207 µg/L, while up to 68,000 µg/L anatoxins were present in lake samples containing large amounts of mat material. The findings of this study are extremely important as they underscore the role of less-studied benthic cyanobacteria in the production of potent toxins, such as the neurotoxic anatoxins. 

Several studies reported results on new or less common cyanobacteria metabolites and cyanotoxin producers. Kust et al. [14] applied a molecular networking and dereplication approach in high-resolution mass spectrometry data using the open global natural product social networking (GNPS) web platform to putatively identify a wide range of cyanopeptides from eutrophic fishponds in the Czech Republic. Forty peptides belonging to the groups of anabaenopeptins, microcystins, cyanopeptolins, microginins, cyanobactins, radiosumins, planktocyclins and epidolastatins were identified. Zervou et al. [15] reported the occurrence and structural variety of anabaenopeptins in cyanobacterial blooms and cultured strains from Greek freshwaters using LC-MS/MS. Thirteen structures of anabaenopeptins were annotated based on interpretation of fragmentation spectra, including three structures not reported before. Cordeiro et al. [16] screened 157 strains from the Azorean Bank of Algae and Cyanobacteria (BACA) for cyanotoxin production (microcystins, saxitoxins and cylindrospermopsins) using qPCR, LC-MS/MS and 16S rRNA phylogenetic analysis. Cyanotoxin-producing genes were amplified in 13 strains, and 4 were confirmed as toxin producers by LC-MS/MS. Two nostocalean strains, possibly belonging to a new genus, were identified as new cylindrospermopsin producers, as they were positive for *cyrB* and *cyrC* genes and the presence of cylindrospermopsin was further confirmed by LC-MS/MS. 

Two papers reported effects of nutrient and climate factors on the proliferation of cyanobacteria and the production of cyanotoxins. Barnard et al. [17] investigated the role of phosphorus and nitrogen limitation on microcystin and anatoxin production from *Microcystis* spp. and *Planktothrix* spp. in Western Lake Erie. The results showed the importance of reducing both nitrogen and phosphorus to limit cyanotoxin and cyanobacterial biomass production. Le Moal et al. [18] analyzed 13 years of eutrophication and climatic data of Lac au Duc, one of the largest shallow water bodies in Brittany, Western France, which is used as recreational and drinking water reservoir. Analysis showed interannual variability of cyanobacterial composition, with dominant species shifting from *Planktothrix agardhii* towards *Microcystis* sp. and then *Dolichospermum* sp. due to climatic pressures and nitrogen limitation. 

Paleolimnological studies based on analysis of sediment cores for cyanobacteria and cyanotoxins, can contribute historic data on the prevalence of toxic cyanobacterial blooms. Weisbrod et al. [19] explored the spatial variability and historical cyanobacterial composition in sediment cores from Lake Rotorua in the South Island of New Zealand, focusing on the abundance of *Microcystis*, *mcyE* gene copy numbers and microcystins. The results showed that toxin producing *Microcystis* blooms are a relatively recent phenomenon in Lake Rotorua, initiated after the 1950s. In addition, results indicated that a single sediment core sampling used by most paleolimnological studies in small to medium-sized lakes can capture dominant microbial communities.

The Special Issue includes three review papers that present emerging areas of toxic cyanobacteria and cyanotoxins research. Metcalf and Codd [20] reviewed and discussed cases were cyanobacteria and cyanotoxins co-occurred with additional hazards such as algal toxins, microbial pathogens, metals, pesticides and microplastics. The authors discussed challenges in assessment of toxicity in such cases and identified further research needs in this field. Sundaravadivelu et al. [21] reviewed the current methodologies for the analysis of freshwater cyanotoxins and prymnesins with emphasis in samples other than water. The authors discussed their limitations, especially with respect to accurate quantitation and structural confirmation of various cyanotoxins, where mass spectrometric techniques are advantageous as they can potentially be applied for detection and unambiguous identification of multiple toxins. Lastly, Monteiro et al. [22] reviewed the existing knowledge on the less-studied, structurally diverse cyclic hexapeptides anabaenopeptins that are increasingly detected in freshwaters in elevated concentrations and possibly play important roles in aquatic ecosystems.
